# Phenylalanine Ammonia-Lyase GhPAL9 Confers Resistance to Verticillium Wilt in Cotton

**DOI:** 10.3390/ijms26114983

**Published:** 2025-05-22

**Authors:** Chuanzong Li, Guoshuai Zhang, Guanfu Cheng, Qi Wang

**Affiliations:** 1 Biotechnology Research Institute, Chinese Academy of Agricultural Sciences, Beijing 100081, China; lichuanzong1994@163.com; 2 Institute of Plant Protection, Xinjiang Academy of Agricultural Sciences, Urumqi 830091, China; 17325221793@163.com (G.Z.); cgfyouxi@163.com (G.C.); 3 Tianjin Key Laboratory of Crop Genetics and Breeding, Tianjin Crop Research Institute, Tianjin Academy of Agricultural Sciences, Tianjin 300384, China

**Keywords:** cotton, Verticillium wilt, *GhPAL9*, lignin, resistance

## Abstract

Verticillium wilt (VW), induced by the soil-borne fungus *Verticillium dahliae*, represents a significant threat to global cotton production. Phenylalanine ammonia-lyase (PAL) is an essential enzyme in lignin metabolism that helps plants defend themselves against pathogenic fungal. Nonetheless, its role in imparting resistance to *V. dahliae* in cotton required further investigation. This study identified the *GhPAL9* (GH_D04G1247) as a crucial gene in cotton resistance to *V. dahliae* via RNA-seq analysis, demonstrating significant upregulation in the resistant variety Xinluzao84. Bioinformatics analysis revealed the conserved evolutionary relationship of GhPAL9 with PAL homologs across various species and highlighted stress-responsive cis-elements in its promoter region. The expression of *GhPAL9* was rapidly activated in roots, stems, and leaves following infection with *V. dahliae*, peaking between 2 and 8 h post inoculation (hpi). Silencing *GhPAL9* through virus-induced gene silencing (VIGS) technology intensified disease symptoms, elevated relative fungal biomass, and diminished lignin accumulation, thereby affirming its function in cotton resistance to *V. dahliae*. The overexpression of *GhPAL9* in *Arabidopsis* improved resistance to *V. dahliae*, and the OE-*GhPAL9* transgenic lines demonstrated reduced disease severity and diminished relative fungal biomass. The results gave us new information about how VW resistance at the molecular level, which established that *GhPAL9* acted as a positive regulator to increase resistance to VW via lignin accumulation.

## 1. Introduction

Cotton stands out as a major cash crop on a global scale, holding strategic significance in the global agricultural economy. Cotton cultivation encounters various challenges, notably Verticillium wilt (VW), which is caused by *Verticillium dahliae* and *Verticillium albo-atrum* and is especially destructive. This soil-caused vascular disease, commonly known as the “cancer” of cotton, significantly affects cotton yield [1,2]. The primary mechanism through which *V. dahliae* exerts its pathogenicity is the blockage of xylem vessels and the production of toxins. When the fungus invades the plant, its mycelium obstructs the xylem vessels, thereby disrupting the normal transport of water and nutrients throughout the plant [3]. Meanwhile, the vigorous transpiration and respiration in the aerial parts of the plant lead to a significant water imbalance, resulting in symptoms such as leaf wilting and yellowing, and ultimately causing plant death. In the context of toxin production, histological evidence suggests that leaf necrosis is primarily induced by the action of toxins [4,5]. Current research suggests that these two pathogenic mechanisms may act in concert to cause VW [6]. The pathogen invaded cotton roots, subsequently colonizing the vascular system and causing symptoms including leaf wilting, browning of vascular bundles, and, in severe instances, total plant death, leading to significant economic losses for the cotton industry [7,8].

Conventional breeding techniques for disease resistance were restricted by the scarcity of resistance gene resources and barriers to interspecific hybridization [9]. The identification of disease resistance genes, combined with biotechnological breeding methods, had emerged as a crucial strategy for enhancing cotton resistance [10]. Contemporary investigations into cotton disease resistance genes predominantly emphasize genes linked to signaling pathways, including the *WRKY*, *MYB*, and *NAC* transcription factor families [11,12,13,14,15,16]. Genes related to hormone metabolism pertinent to jasmonic acid (JA), salicylic acid (SA), and ethylene (ET) pathways [17,18,19], and enzyme-encoding genes such as chitinase, peroxidase, and those associated with the phenylalanine pathway [20,21,22]. Furthermore, research has investigated immune receptor genes, antitoxin protein genes, and ribosomal proteins [23,24,25]. The characterization and mechanistic analysis of these genetic resources establish a theoretical basis for molecular breeding focused on improving disease resistance in cotton [26].

Phenylalanine ammonia-lyase (PAL) is an enzyme that regulate the phenylpropanoid metabolic pathway in plants, with multiple *PAL* genes identified in species including *Arabidopsis thaliana*, *Sorghum bicolor*, *Oryza sativa*, and *Triticum aestivum* [27,28]. Investigations into *PAL* genes have predominantly concentrated on their functions in resistance to abiotic stresses, such as cold, heat, salinity, and drought. Research on *PAL*-mediated resistance to biotic stresses has been performed in crops including *O. sativa* and *Zea mays*. In rice, the knockdown of *OsPALs* significantly diminished resistance to the brown planthopper, while the overexpression of *OsPAL8* in susceptible rice cultivars substantially increased resistance to this pest [29]. Moreover, *OsPAL4* has been associated with rice defense mechanisms against bacterial wilt, rice blast, and rice sheath blight [30]. *PAL* genes in maize have been linked to resistance against maize sheath blight [31]. Nevertheless, investigations concerning the function of *PAL* genes in biotic stress resistance in cotton are still sparse.

In a previous study, we performed a transcriptomic analysis of the dominant upland cotton cultivars in Xinjiang, namely the resistant variety Xinluzao84 and the susceptible variety J8031, subsequent to infection with *V. dahliae*. Bioinformatics analyses revealed numerous differentially expressed genes (DEGs), with *GhPAL9* showing significantly elevated expression in the resistant variety. This study further corroborated that *GhPAL9* expression was stimulated by *V. dahliae* infection. Virus-induced gene silencing (VIGS) of *GhPAL9* markedly diminished cotton resistance to *V. dahliae* and resulted in decreased lignin accumulation. Moreover, the overexpression of *GhPAL9* in *Arabidopsis* provided increased resistance to *V. dahliae* relative to the wild-type (WT) plants. This study highlights the essential function of *GhPAL9* in cotton defense *V. dahliae* and demonstrates that *GhPAL9* bolsters cotton resistance through the facilitation of lignin accumulation.

## 2. Results

### 2.1. Identification of the GhPAL9 Gene in Cotton

**Figure 1:**
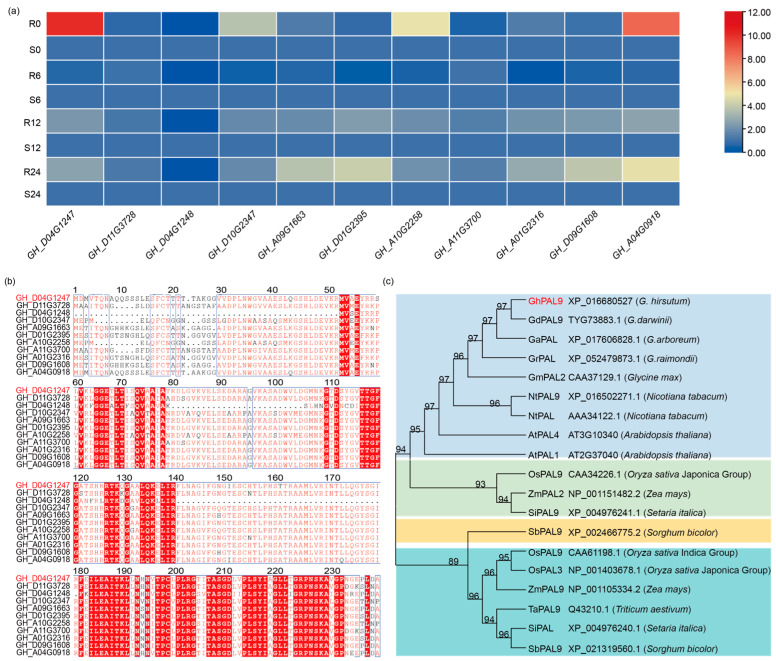
Bioinformatics analysis of *GhPAL9*. (**a**) Expression levels of 11 *GhPAL* genes, derived from RNA-seq data, are shown in the heat map. Blue-to-red color gradient of scale bar represents low expression levels and high expression levels, respectively. Red-marked GH_D04G1247 was selected for further investigation. (**b**) A comparative analysis of the protein sequences of GhPALs (partial); White letters on a red square represent prevalent sequence residues, while red is used to depict analogous residues, blue boxes indicate conserved sequences. (**c**) Phylogenetic analysis of GhPAL9 and PALs from various species, red indicates upland cotton GhPAL9 protein; MEGA X program was used to build the neighbor-joining phylogenetic tree.

We examined RNA-seq data (PRJNA1245489) from the resistant cotton variety Xinluzao84 and the susceptible variety J8031, identifying numerous differentially expressed *GhPAL* genes. GH_D04G1247 (*GhPAL9*) demonstrated markedly elevated expression in the resistant variety (Figure 1a). The complete cDNA sequence of *GhPAL9* comprises 2166 nucleotides and has 721 amino acids and coded for a protein. A multiple sequence alignment of GhPAL proteins was conducted to evaluate sequence conservation, indicating that GhPAL9 exhibited significant sequence identity with other GhPAL family members in *Gossypium hirsutum* L. (Figure 1b). A phylogenetic analysis was performed to examine the evolutionary relationships of GhPAL9 by comparing it with PAL9 from other species, such as rice, wheat, and maize. The findings categorized these genes into four distinct subgroups, demonstrating that PAL9 was evolutionarily conserved. Furthermore, GhPAL9 was identified as being most closely related to GdPAL9 (Figure 1c). An analysis of the three-dimensional structural model of the GhPAL9 protein indicated that it primarily comprises α-helical structures, implying potential functional consequences for enzyme activity and stability (Figure S1a). Additionally, to investigate the regulatory mechanisms controlling *GhPAL9* expression, we examined its promoter region for cis-regulatory elements. The findings revealed several components linked to responses to diverse stimuli, encompassing hormone-related elements, transcription factor binding sites, and cis-elements associated with stress responses. The findings indicated that *GhPAL9* expression was probably modulated by a combination of external stress, endogenous hormones, and transcriptional regulators, underscoring its potential involvement in stress response pathways (Figure S1b).

### 2.2. The Expression of GhPAL9 Was Upregulated in Response to V. dahliae Infection

**Figure 2:**
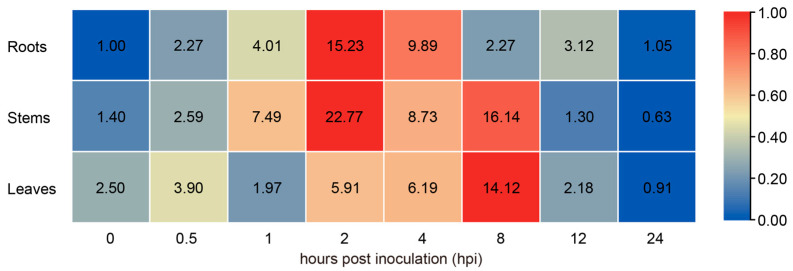
Expression pattern of *GhPAL9* in various cotton tissues inoculated with *V. dahliae*. Quantification of *GhPAL9* expression via RT-qPCR. Tissues of two-week-old seedlings were collected at 0–24 hpi for total RNA extraction. Blue-to-red color gradient of the scale bar represents low expression levels and high expression levels, respectively.

To elucidate the role of *GhPALs* in disease responses, we analyzed the expression pattern of *GhPALs* in cotton roots following inoculation with *V. dahliae* (Figure S2). The study found that the *GhPAL9* gene was significantly induced, and to further elucidate the role of *GhPAL9*, we analyzed the expression pattern of *GhPAL9* in various cotton tissues. Following inoculation with *V. dahliae*, *GhPAL9* expression was significantly up regulated in roots, stems, and leaves tissues, particularly in stems. The expression of *GhPAL9* was markedly up regulated in roots and stems, peaking at 2 hpi, while the highest expression in leaves occurred at 8 hpi, followed by a gradual decline. These results indicated that *GhPAL9* was implicated in cotton resistance to *V. dahliae* infection (Figure 2).

### 2.3. Silencing GhPAL9 Expression Decreased Cotton Resistance to V. dahliae

**Figure 3:**
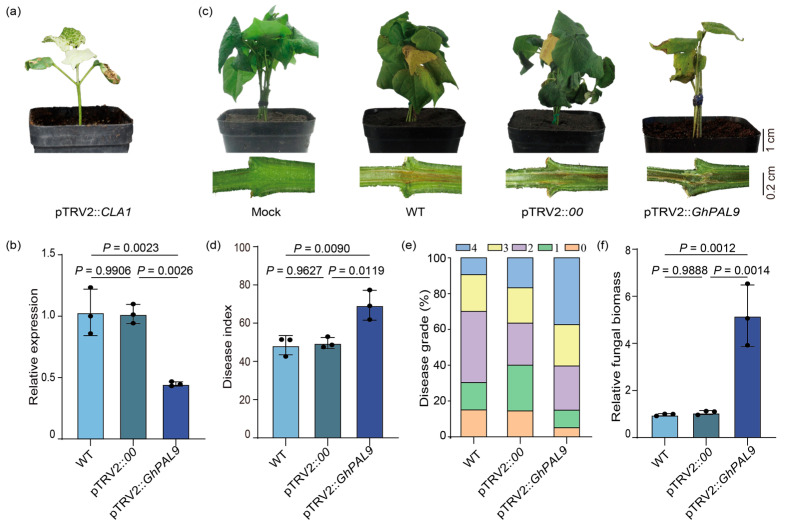
Knockdown of *GhPAL9* attenuated cotton resistance to *V. dahliae*. (**a**) Effect of pTRV2::*CLA1* infection on phenotype of cotton seedlings; (**b**) RT-qPCR analysis of *GhPAL9* expression in silenced and control plants. For total RNA extraction, we took leaves from seedlings that were two weeks old. (**c**) Cotton disease symptoms at 14 days post-infection and (**d**) cotton disease index at 14 days post-infection; error bars show the SD from three separate experiments; (**e**) Values of 0–4 represent disease severity; (**f**) qRT–PCR was used to quantify relative biomass of *V. dahliae* in infected stems. Reference genes used were *GhUBQ7* and *V. dahliae β-tubulin*. There were three biological replicates, and error bars show the SD of those. Error bars denote mean ± SD, ((**b**,**d**,**f**), *n* = 3 biological replicates), dots are individual measurements for the experimental group, and *p*-values are from one-way ANOVA.

To evaluate the function of *GhPAL9* in the response to *V. dahliae*, we utilized VIGS technology to inhibit the expression of *GhPAL9*. pTRV2::*CLA1* caused cotton to produce a photobleaching phenotype as a positive control (Figure 3a); the WT, pTRV2::*00*, and pTRV2::*GhPAL9* cotton were collected to assess the transcript levels of *GhPAL9*. The expression of *GhPAL9* was significantly decreased in pTRV2::*GhPAL9* plants with 70% inhibition value relative to the control (Figure 3b), whereas the expression of other *GhPAL* genes did not show a significant decrease (Figure S3), indicating that *GhPAL9* was silenced in pTRV2::*GhPAL9* but not other *GhPAL* genes. Water treatment served as a control (Mock), while WT, pTRV2::*00*, and pTRV2::*GhPAL9* cotton were infected with *V. dahliae*, and the phenotypes were assessed two weeks later. All inoculated cotton displayed the characteristic phenotype of wilting, yet the pTRV2::*GhPAL9* cotton demonstrated more pronounced symptoms of leaf wilting and defoliation, along with a more significant browning of the stems (Figure 3c). Silenced cotton had a higher disease index than the control (Figure 3d), with an elevated percentage of grade 4 diseased cotton (Figure 3e), and relative fungal biomass was much higher in silenced cotton than in WT (Figure 3f). The findings suggested that *GhPAL9* knockdown decreased *V. dahliae* resistance in cotton.

### 2.4. Decreased GhPAL9 Expression Affected Lignin Accumulation

**Figure 4:**
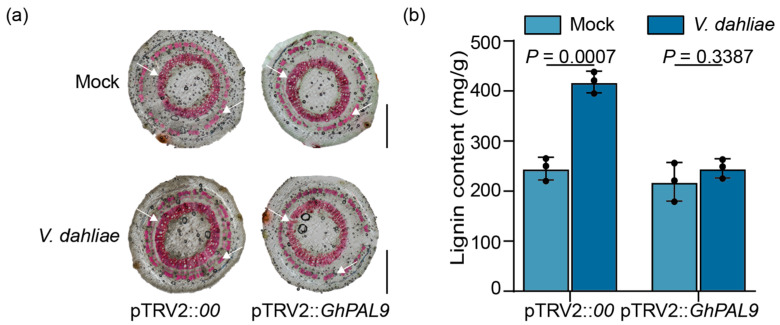
Silencing of *GhPAL9* reduced lignin accumulation in cotton. (**a**) Examination of lignin buildup in stem sections of pTRV2::*00* and *GhPAL9*-silenced cotton at 2 hpi using histochemical staining with either water (Mock) or *V. dahliae*, white arrows indicate sites of stained lignin deposition. (**b**) Measurements of lignin content in roots of pTRV2::*00* and *GhPAL9*-silenced cotton at 2 hpi treated with water (Mock) or *V. dahliae*. Scale bars measure 0.05 cm. Error bars denote mean ± SD, ((**b**), *n* = 3 biological replicates), dots are individual measurements for the experimental group, and *p*-values are from the *t*-test.

To ascertain the function of *GhPAL9* in cotton resistance to VW through its influence on lignin, we conducted histochemical staining and lignin quantification in pTRV2::*00* and pTRV2::*GhPAL9* cotton. When subjected to water treatment, pTRV2::*00* and pTRV2::*GhPAL9* cotton showed no statistically significant differences in lignin staining area or content. Upon infestation with *V. dahliae*, the area of lignin staining was markedly pronounced, and the lignin content was significantly increased in the control group (Figure 4a). Conversely, the lignin content in pTRV2::*GhPAL9* cotton exhibited only a marginal increase without any significant difference, aligning with the staining results (Figure 4b). The results demonstrated that *GhPAL9* serves as a regulatory gene for lignin biosynthesis in response to *V. dahliae* infection, with silencing of its expression resulting in reduced lignin accumulation.

### 2.5. GhPAL9 as a Positive Regulator of Plant Resistance Against V. dahliae in Arabidopsis

**Figure 5:**
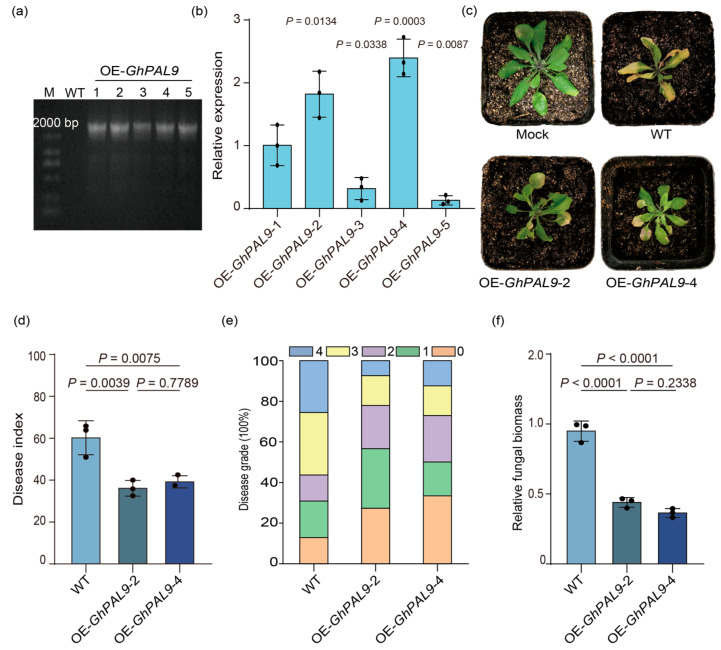
Increased resistance to *V. dahliae* in *Arabidopsis* overexpressing *GhPAL9*. (**a**) PCR amplification was used to detect positive transformants. M: maker, DL2000 Plus DNA Marker (MD101, Vazyme); (**b**) *GhPAL9* expression in different OE-*GhPAL9* lines using RT-qPCR. For total RNA extraction, seedlings’ leaves were collected at three weeks of age. An internal control was provided by *Atactin2*. Error bars show SD for three separate experiments; (**c**) *Arabidopsis* disease symptoms at 7 days after inoculation; (**d**,**e**) *Arabidopsis* disease index and values of 0–4 represent disease grade at 7 days after inoculation; (**f**) Using RT-qPCR, the relative biomass of *V. dahliae* in infected *Arabidopsis* was quantified. Reference genes used were *AtActin2* and *V. dahliae β*-tubulin, and error bars denote mean ± SD, ((**b**), *n* = 3 biological replicates), dots are individual measurements for the experimental group, and *p*-values are from the t-test. Error bars denote mean ± SD, ((**d**,**f**), *n* = 3 biological replicates), dots are individual measurements for the experimental group, and *p*-values are from one-way ANOVA.

To validate the disease resistance impact of *GhPAL9*, the gene was overexpressed in *Arabidopsis* through *Agrobacterium*-mediated flower-dipping, resulting in five T_2_ pure transgenic lines (Figure 5a). Among these, OE-*GhPAL9*-2 and OE-*GhPAL9*-4, exhibiting elevated expression levels as determined by RT-qPCR analysis, were chosen for subsequent experimentation (Figure 5b). Seven days post-inoculation with *V. dahliae*, characteristic symptoms of wilt were prominently observed in the infected WT plants, whereas they were significantly less pronounced in the OE-*GhPAL9*-2/4 transgenic lines (Figure 5c). The results of the statistical analysis showed that the average disease index and the relative fungal biomass of the OE-*GhPAL9*-2/4 transgenic lines were a lot lower than that of the WT (Figure 5d-f). Based on the evidence, we concluded that the overexpression of *GhPAL9* could enhance the resistance of *Arabidopsis*.

## 3. Discussion

Identifying and screening resistance genes was crucial for understanding the mechanisms of disease resistance in cotton. This study analyzed RNA-seq data from the resistant cotton variety Xinluzao84 and the susceptible variety J8031, both inoculated with *V. dahliae*, to identify potential resistance genes against VW. The findings indicated that multiple *GhPAL* genes were differentially expressed, with *GhPAL9* showing a markedly elevated expression in the resistant variety. Analysis of gene expression patterns revealed that *GhPAL9* expression in roots and stems was markedly upregulated after *V. dahliae* infection, peaking at 2 hpi, whereas its expression in leaves peaked at 8 hpi. The findings indicated that *GhPAL9* may be pivotal in the cotton defense mechanism against *V. dahliae*.

*PAL* facilitated the transformation of phenylalanine into trans-cinnamic acid, which served as a precursor for secondary metabolites like lignin [32,33]. Recent studies had shown that *PAL* genes were essential for providing resistance to biotic stresses in multiple crops. In *Arabidopsis*, the quadruple mutants (*pal1*, *pal2*, *pal3*, *pal4*) exhibited significantly reduced levels of SA and increased susceptibility to a virulent strain of the bacterial pathogen *Pseudomonas syringae* [34]. Similarly, in *CaPAL1*-silenced pepper plants infected with *Xanthomonas campestris* pv. *vesicatoria* (Xcv), the accumulation of reactive oxygen species (ROS), hypersensitive cell death, and induction of PAL activity were all significantly compromised [35]. In contrast, overexpression analyses demonstrated that *GmPAL2.1* enhanced resistance to *Phytophthora sojae* in transgenic soybean plants. Moreover, in *GmPAL2.1*-overexpressing transgenic soybean, the levels of daidzein, genistein, and SA were markedly increased, along with a significant rise in the relative content of glyceollins [36]. Recent studies on *PAL* genes in cotton revealed that the majority possess stress-responsive cis-elements and can be activated by multiple stresses. Under drought and salt stress conditions, elevated expression of the *GhPAL* gene significantly enhanced cotton tolerance [37]. Conversely, silencing the lignin biosynthesis-related gene *GhPAL* (*GH*_A04G0918) in cotton via VIGS led to a reduction in lignin content and weakened resistance to lodging [38]. Meanwhile, transient overexpression of the transcription factor *GhMYB18* in cotton activated the phenylalanine signaling pathway, which notably boosted the activity of GhPAL and thereby strengthened cotton tolerance to aphid feeding [39]. Similarly, the resistant cotton variety Pima-S6, when confronted with Fusarium wilt, bolstered root phenylpropanoid metabolism and disease resistance by upregulating the activity of GhPAL2 [40].

Nonetheless, conclusive research regarding the role of *GhPAL* genes in augmenting cotton resistance to *V. dahliae* is scarce. VIGS was a powerful technique for gene silencing that leverages plant viruses, which can specifically target degrades cellular mRNAs complementary to the target gene sequence, effectively silencing the gene [41,42]. This study demonstrated that transient silencing of *GhPAL9* through VIGS markedly diminished cotton resistance to *V. dahliae*, while overexpression of *GhPAL9* resulted in improved resistance in *Arabidopsis*. Although *GhPAL9* and *GH_A04G0918* were members of the same PAL family, they have undergone functional differentiation to meet distinct biological needs. *GH_A04G0918* was likely to be predominantly involved in lignin deposition associated with developmental processes, while *GhPAL9* was rapidly activated in response to pathogen-related signals, further promoting lignin synthesis associated with disease resistance. The results showed that *GhPAL9* was one of the most important positive regulators in the cotton defense response to *V. dahliae*.

Lignin, a complex aromatic polymer, bolstered plant resistance by strengthening cell wall structures, engaging in signal transduction pathways, and impeding the proliferation of pathogenic fungal [43]. The transcription factor MYB30 in rice facilitates lignin accumulation by regulating genes involved in lignin biosynthesis [44]. In cotton, *GhLAC15* had been demonstrated to markedly improve resistance to VW by augmenting the accumulation of arabinose and xylose in lignin and cell walls [45]. This study demonstrated that transient silencing of *GhPAL9* significantly decreased lignin accumulation, thereby reinforcing the hypothesis that *GhPAL9* enhanced cotton resistance to VW by regulating lignin biosynthesis. The exact molecular mechanism through which *GhPAL9* regulated lignin accumulation, influenced cell wall mechanical strength, and ultimately imparted resistance to VW necessitates additional research.

## 4. Materials and Methods

### 4.1. Growth of Plant Material and V. dahliae Cultures

This research employed Coker312 (R15), *A. thaliana* Columbia-0 (Col-0), and *V. dahliae* strain *Vd*991 as experimental subjects. Cotton and *Arabidopsis* plants were grown under regulated conditions at 28 °C and 21 °C, respectively, with an 8 h light and 16 h dark photoperiod and 60% relative humidity. The *V. dahliae* strain *Vd*991 was initially cultivated on PDA medium (potato 200 g/L, glucose 20 g/L, agar 20 g/L) and subsequently transferred to CM liquid culture (yeast extract 6 g/L, casein acid hydrolysate 6 g/L, sucrose 10 g/L) at 28 °C, 200 rpm [46].

### 4.2. Bioinformatic Analysis

The protein sequence of GhPAL9 in cotton was aligned with the protein sequences of PAL in *A*. *thaliana*, *Z*. *mays*, and *O*. *sativa* using MEGA software (X, Sudhir Kumar, Philadelphia, America), and a phylogenetic tree was constructed using the neighbor-joining method. The multiple sequence alignment of GhPAL family members was conducted applying the ESPript3.0 web server (https://espript.ibcp.fr/ESPript/ESPript/) (accessed on 15 February 2025). The three-dimensional conformation of GhPAL9 was forecasted utilizing the Swiss Model web server (https://swissmodel.expasy.org/) (accessed on 17 February 2025). The promoter sequence of *GhPAL9* was examined for cis-acting elements using the Plant CARE database (https://bioinformatics.psb.ugent.be/webtools/plantcare/html/) (accessed on 20 February 2025), and the findings were visualized with TBtools software (2.007, Chengjie Chen, Guangzhou, China).

### 4.3. Gene Expression Analysis

Cotton seedlings were inoculated with *V. dahliae* spore suspension (1 × 10^7^ cfu/mL), and roots, stems, and leaves tissues were harvested at 0, 0.5, 1, 2, 4, 8, 12, and 24 h post inoculation (hpi). Total RNA was extracted with the RNA Easy Fast Kit (Tiangen, Beijing, China), and complementary DNA (cDNA) was synthesized utilizing the HiScript IV All-in-One Ultra RT SuperMix for qPCR (Vazyme, Nanjing, China). The housekeeping gene *GhUBQ7* served as an internal reference, and the RT-qPCR primers for *GhPAL9* are detailed in Table S1. Quantitative reverse transcription polymerase chain reaction (RT-qPCR) was performed utilizing the ChamQ SYBR qPCR Master Mix (Vazyme, Nanjing, China) on an ABI 7500 Fast Real-Time PCR system (Applied Biosystems, Foster City, CA, USA). Relative gene expression levels were determined utilizing the 2^−ΔΔCt^ methodology.

### 4.4. Virus-Induced Gene Silencing (VIGS)

VIGS is a powerful reverse genetics technology in plants, employing modified viral vectors to elicit sequence-specific post-transcriptional gene silencing. A 300 bp sequence-specific interference fragment within the coding sequence (CDS) of *GhPAL9* was identified using the SGN VIGS Tool (https://vigs.solgenomics.net/) (accessed on 2 February 2025) and amplified with 2× Phanta Flash Master Mix (Dye Plus). The fragment was ligated into the pTRV2 vector to create the pTRV2::*GhPAL9*, which was subsequently transformed into *Agrobacterium* tumefaciens strain GV3101. *Agrobacterium* strains containing pTRV1, pTRV2::*00*, pTRV2::*CLA1*, and pTRV2::*GhPAL9* were cultivated in LB medium augmented with kanamycin (100 µg/mL) and rifampicin (50 µg/mL) at 28 °C with agitation at 220 rpm. Once the OD_600_ reached 0.6–0.8, the cultures were transferred to new LB medium that had been supplemented with kanamycin, rifampicin, MES, and AS. They were then left to incubate. The cultures were subsequently centrifuged at 6000 rpm for 5 min and resuspended in ddH_2_O. *Agrobacterium* strains harboring pTRV2::*00*, pTRV2::*CLA1*, and pTRV2::*GhPAL9* were combined with pTRV1 in a 1:1 ratio. Cotton seedlings exhibiting fully expanded cotyledons were chosen for infiltration and incubated in darkness at 25 °C for 48 h, utilizing pTRV2::*CLA1* as a positive control. Inoculation with pTRV2::*00* and pTRV2::*GhPAL9* was conducted when pTRV2::*CLA1* plants displayed photobleaching and the disease index, fungal biomass was measured at 14 dpi.

### 4.5. Pathogenic Assay and Determination of Fungal Biomass

*V. dahliae* was cultivated in CM medium for 48 h, and the spore suspension was quantified with a hemocytometer and adjusted to a final concentration of 1 × 10⁷ cfu/mL. Cotton roots were submerged in the suspension for 5 min, and disease severity was evaluated 14 days after inoculation. The disease index (DI) was computed utilizing the formula: DI (%) = [(∑Disease grades × Number of infected plants)/(Total checked plants × 4)] × 100 [47].

Root tissues from cotton and *Arabidopsis* plants were collected, and genomic DNA was isolated utilizing the Genomic DNA Extraction Kit (Tiangen, Beijing, China). The ITS region of *V. dahliae* ribosomal RNA genes were amplified utilizing *Vd*-F/*Vd*-R primers (Table S1) [48]. The housekeeping genes *GhUBQ7* (from cotton) and *Atactin2* (from *Arabidopsis*) functioned as internal references, with the relevant primers detailed in Table S1. Fluorescence-based qPCR was conducted as outlined in Section 4.3, and relative fungal biomass was quantified employing the 2^−ΔΔCt^ method.

### 4.6. Histochemical Staining and Quantification of Lignin

The total lignin accumulation in cotton stems was evaluated utilizing a resorcinol staining kit (Solarbio, Beijing, China). Stem segments from pTRV2::*00* and pTRV2::*GhPAL9* plants were sliced to a thickness of 200 µm and immersed in a 1% ethanol resorcinol solution for 10 min. After rinsing with ddH₂O, the distribution and intensity of lignin staining were analyzed and documented using a microscope (Thermo Fisher Scientific, Waltham, MA USA). The lignin content was measured using a lignin assay kit (Solarbio, Beijing, China).

### 4.7. Arabidopsis Transformation and Identification of Positive Transgenic Plants

*Agrobacterium* strain GV3101 was transfected with the CDS of *GhPAL9* after it was cloned into the overexpression vector pCAMBIA2300. *Agrobacterium* harboring the OE-*GhPAL9* plasmid was cultivated to OD_600_ = 0.6–0.8, next collected by spinning at 5000 rpm for 20 min, and then mixed with 100 mL of transformation buffer (5% sucrose and 0.05% Silwet L-77) to OD_600_ = 0.8–1.0. *Arabidopsis* floral buds were immersed in the suspension for 1 min and subsequently incubated in darkness for 16 to 24 h. Three days later, a subsequent infestation was executed to improve transformation efficiency. T₀ generation seeds were cultured in a 1/2 MS screening medium supplemented with kanamycin resistance. Following several rounds of stringent selection, T₂ transgenic lines were successfully obtained. Subsequently, total DNA was extracted from both WT and transgenic plants. The complete CDS sequences of *GhPAL9* were amplified using 2 × Rapid Taq Master Mix (P222, Vazyme) with specific detection primers (Table S1) and verified by Sanger sequencing. The PCR system components were as follows: 25 μL of 2× Rapid Taq Master Mix, 19 μL ddH_2_O, 2 μL GhPAL9-F (10 μM), 2 μL GhPAL9-R (10 μM), and 2 μL DNA. The PCR program comprised a cycle at 95 °C for 3 min, 95 °C for 15 s, 62 °C for 15 s, and 72 °C for 15 s, followed by 35 additional cycles of 95 °C for 15 s, 62 °C for 15 s, and 72 °C for 15 s, and finally 72 °C for 5 min.

### 4.8. Statistical Analysis

Statistical analysis was performed using Graphpad Prism software (8.0.0, San Diego, CA, USA), and provided exact *p*-values. The *t*-test was used to compare the mean differences between two groups of independent samples, and one-way ANOVA was used to compare the mean differences between more than two groups of independent samples.

## 5. Conclusions

The *GhPAL9* was crucial in regulating resistance to VW in this study. Following *V. dahliae* infection, the expression of *GhPAL9* was elevated in stems, roots, and leaves, with peak expression in roots and stems occurring at 2 hpi, and peak expression in leaves at 8 hpi. According to the VIGS analysis, *GhPAL9* silencing reduced lignin accumulation and cotton resistance to *V. dahliae*. Increasing *Arabidopsis* resistance to *V. dahliae* was achieved through the overexpression of *GhPAL9*. In summary, our research demonstrated that *GhPAL9* significantly influences cotton resistance to *V. dahliae*, providing a genetic foundation for the development of highly resistant cotton varieties.

## Data Availability

The original contributions presented in the study are publicly available. This data can be found here: NCBI, PRJNA1245489.

## Supplementary Materials

The following supporting information can be downloaded at: https://www.mdpi.com/article/10.3390/ijms26114983/s1.
