# Supplementary material for: Phenylalanine Ammonia-Lyase GhPAL9 Confers Resistance to Verticillium Wilt in Cotton
Source: Int J Mol Sci. 2025 May 22;26(11):4983. doi: 10.3390/ijms26114983 (PMC12154288; doi:10.3390/ijms26114983)
Supplement: Supplementary file 1 [file ijms-26-04983-s001.zip › ijms-3599560-supplementary.pdf]

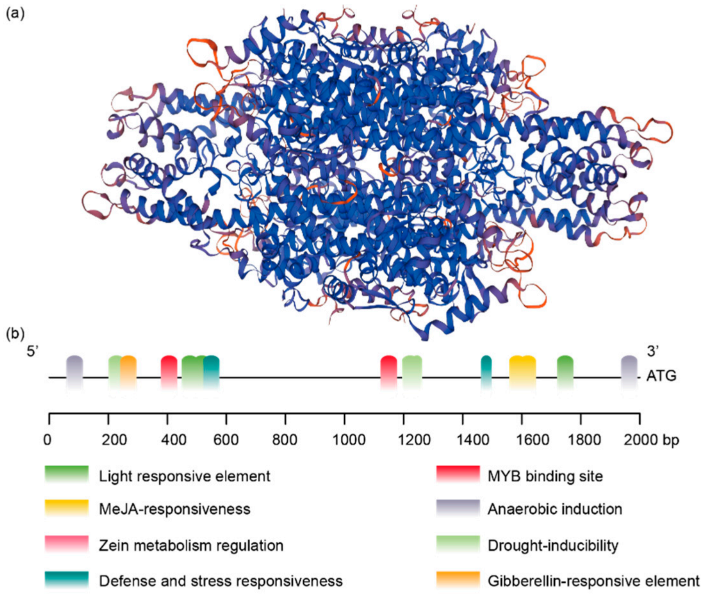

Figure S1. Bioinformatics analysis of *GhPAL9*. (a) The three-dimensional structure of GhPAL9 was generated via SWISS-MODEL websites; (b) Using Plant Care websites to look at cis-acting elements in the *GhPAL9* promoter region.

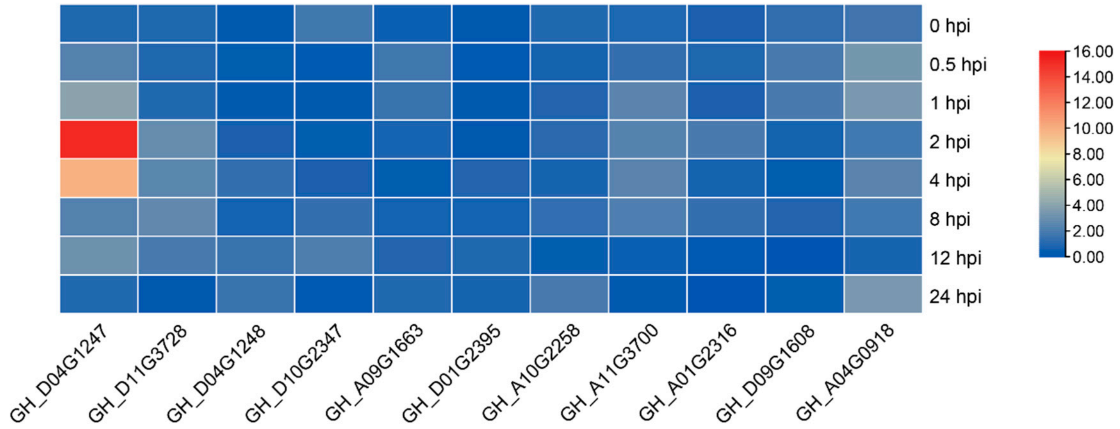

Figure S2. The expression pattern of *GhPALs* in cotton roots inoculated with *V. dahliae*. Quantification of *GhPALs* expression via RT-qPCR. The roots of two-week-old seedlings were collected at 0-24 hpi for total RNA extraction. The blue-to-red color gradient of the scale bar represented low expression levels and high expression levels, respectively.

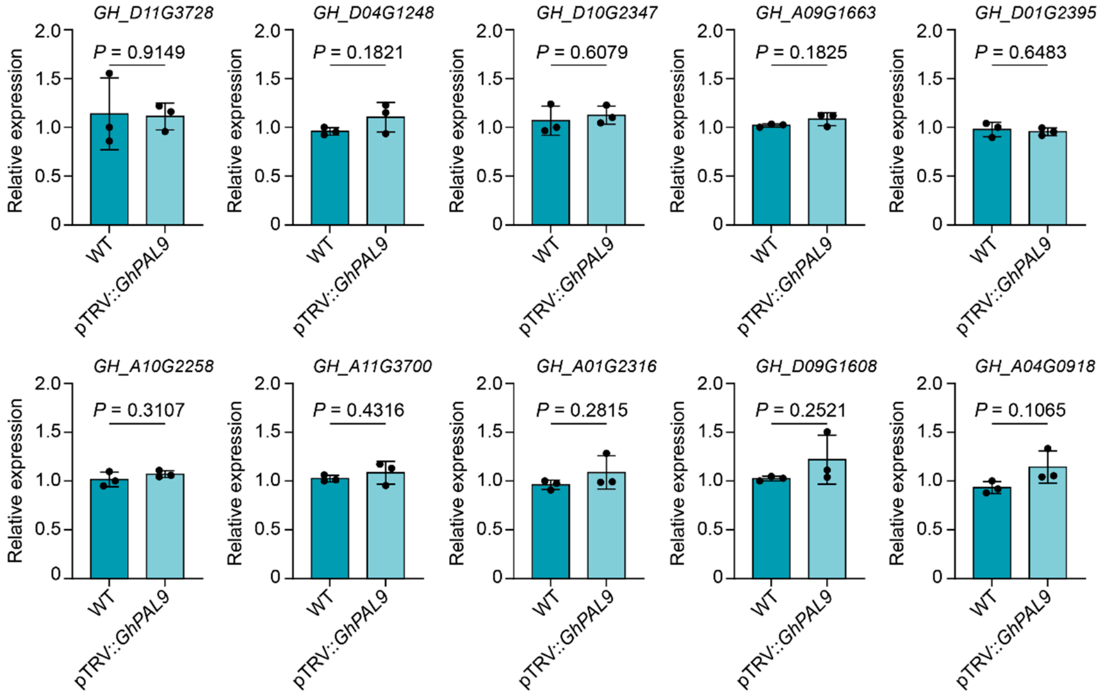

Figure S3. Identification of *GhPAL* genes expression in pTRV2::*GhPAL9* cotton. The expression of different genes in pTRV2::*GhPAL9* was determined by RT-qPCR using *GhUBQ7* as an internal reference gene. Error bars denote mean ± SD, (*n*= 3 biological replicates), dots are individual measurements for the experimental group, and *p* values are from the t-test.

Table S1. The primers used in this study[1]

**Construct names**	**Primer names**	**Primer sequences (5'-3')**	**Product size**
RT-qPCR	qGhPAL9-F	GAAGAACACCGTGAGCCAGA	151 bp
	qGhPAL9-R	GGTAGCACTGCAAGGGTCAT	
	Atactin2-F	AGGTATCGCTGACCGTATGA	148 bp
	Atactin2-R	TACCTGCTGGAATGTGCTGA	
	GhUBQ7-F	AGCTCGGATACGATTGATAACG	166 bp
	GhUBQ7-R	GAAGACGAAGAACAAGGGGAAG	
	Vd-F	CCGCCGGTCCATCAGTCTCTCTGTTTATAC	347 bp
	Vd-R	CGCCTGCGGGACTCCGATGCGAGCTGTAAC	
	GH_D11G3728-F	CGGCAACAACGAGAACAACG	129 bp
	GH_D11G3728-R	GCTGGGTTTCCGTTCTCGAT	
	GH_D04G1248-F	TTCTCAAGTTGCAGCCGTAGCTAAA	148 bp
	GH_D04G1248-R	TGCCTCCAGAATTTTGAACCTAATA	
	GH_D10G2347-F	CCGGCTATCGCGAACAAGAT	178 bp
	GH_D10G2347-R	AGCAATCCAGCAACGGATCA	
	GH_A09G1663-F	GAGAGTGCAAGGGCATCAGT	121 bp
	GH_A09G1663-R	CAGTTAGCAGCCCAGTTCCA	
	GH_D01G2395-F	CAAATGGTGGTGTTGGTGTGC	136 bp
	GH_D01G2395-R	TCACCACCGAGTTTCACCAA	
	GH_A10G2258-F	GAATCCGGCTATCCCGAACA	158 bp

	GH_A10G2258-R	ACTTCCCTGCACACATTGCT	
	GH_A11G3700-F	TTCGGTTGAGAACGGGAACG	133 bp
	GH_A11G3700-R	CTCACCAGGGGACTTCACTT	
	GH_A01G2316-F	AATGGTGGTGTTGGTGTAGTG	135 bp
	GH_A01G2316-R	CTCACCACCGAGTTTCACCAA	
	GH_D09G1608-F	TGCTACCTACCCGTTGATGC	171 bp
	GH_D09G1608-R	ACTGATGCCCTTGCACTCTC	
	GH_A04G0918-F	ATCAGGTTCGCAACCAAATCAA	177 bp
	GH_A04G0918-R	AGCGAACATCAGTTTTCCGATT	
pTRV	V-GhPAL9-F	GTGAGTAAGGTTACC**GAATTC**GACGCTC ACTACAGGTGCCAA	300 bp
	V-GhPAL9-R	CGTGAGCTCGGTACC**GGATCC**GCATTTCCA TTCTCAAGTGTCACC	
PCR	GhPAL9-F	ATGGACATGGTCACTCAAAATGC	2166 bp
	GhPAL9-R	GCAGATTGGAAGAGGGGCACC	

[1]. The restriction recognition sequences of *Bam*H I (GGATCC) and *Eco*R I (GAATTC) within the primers are in bold and underlined. Genes included in qPCR assay are *GhPAL9* (LOC107899351), *GhUBQ7* (LOC107925174), Vd-ITS (MT899267.1), *Atactin2* (AT3G18780).
